# Symptom control among asthmatics with a clinically significant smoking history: a cross-sectional study in Finland

**DOI:** 10.1186/s12890-020-1127-9

**Published:** 2020-04-15

**Authors:** Toni Kiljander, Tuija Poussa, Timo Helin, Antero Jaakkola, Kari Venho, Lauri Lehtimäki

**Affiliations:** 1 Department of Respiratory Medicine, Terveystalo Hospital, Turku, Finland; 2 Stat Consulting, Nokia, Finland; 3 Skin and Allergy Hospital, Helsinki University Central Hospital, Helsinki, Finland; 4 Boehringer-Ingelheim Finland, Helsinki, Finland; 5 Department of Respiratory Medicine, Terveystalo Hospital, Jyväskylä, Finland; 6 Allergy Centre, Tampere University Hospital, Tampere, Finland; 7 Faculty of Medicine and Health Technology, Tampere University, Tampere, Finland

**Keywords:** Asthma, Smoking, Symptom control

## Abstract

**Background:**

Surprisingly little is known about asthma control among asthmatics who smoke. The aim of this cross-sectional study was to investigate asthma symptom control according to the GINA guidelines among asthmatics with a clinically significant smoking history.

**Methods:**

One hundred ninety asthmatics from primary care in Finland were investigated. The patients were current or previous cigarette smokers with a history of 10 or more pack-years. They completed a questionnaire including questions on asthma symptoms and reliever use so that their level of asthma symptom control (well controlled, partly controlled, or uncontrolled) according to GINA could be determined.

**Results:**

Sixty-six (34.7%) patients had their asthma well controlled, 81 (42.6%) had their asthma partly controlled, and 43 (22.6%) had uncontrolled asthma. Current smokers had uncontrolled asthma more often than ex-smokers, OR 2.54 (95% CI 1.25–5.14, *p* = 0.01). Patients with moderate to severe asthma exacerbation during the previous year had uncontrolled asthma more often than patients without an exacerbation, OR 2.17 (95% CI 1.06–4.47, *p* = 0.04), and patients with FEV_1_ <  80% of predicted had uncontrolled asthma more often than patients with FEV_1_ > 80% of predicted, OR 2.04 (95% CI 1.02–4.08, *p* = 0.04).

**Conclusions:**

Asthmatic patients with a clinically significant smoking history often do not have well controlled asthma. Poor asthma symptom control was associated with current smoking status, history of exacerbations and impaired lung function. Therefore, every attempt should be made to help asthmatics who smoke to quit smoking.

## Background

Asthmatic patients smoke roughly as often as the general population. Approximately 20% of asthmatics are smokers [1–3]. Among asthmatic patients, smoking is associated with increased morbidity and mortality, a higher frequency of asthma exacerbations, accelerated decline of lung function, reduced response to inhaled corticosteroids, and increased asthma severity, when compared to non-smoking asthmatics [4, 5].

It has been shown that, smoking, especially currently but also previously, is associated with poorer asthma control levels [6–9]. However, surprisingly little is known about asthma symptom control among asthmatics who smoke.

The aim of this study was to investigate asthma symptom control according to GINA guidelines [10] among primary care asthmatic patients who are either current or ex-smokers and to determine the patient characteristics that may be associated with asthma control level.

## Methods

As part of our previous study assessing the prevalence of undiagnosed asthma-COPD overlap among subjects with asthma and a smoking history, asthmatic subjects with a smoking history of at least 10 pack-years were recruited during their primary health care visits in Finland [11]. The inclusion criteria were age 18–70 years, current or ex-smoker with 10 or more pack-years and doctor-diagnosed asthma. The exclusion criteria were any severe illness, any known pulmonary disease other than asthma, and the use of an inhaled anticholinergic or indacaterol or oral roflumilast (to exclude subjects with COPD).

In addition to measuring spirometry (Medikro, Kuopio, Finland) and collecting information on demographics, smoking history, medical history and medication, a questionnaire (Additional file 1) including questions set according to the GINA guidelines [10] was filled so that the asthma control level (well controlled, partly controlled, or uncontrolled) could be determined.

Patients were considered to have had a moderate to severe asthma exacerbation during the previous year if they had been hospitalized or had used a course of oral corticosteroids for their asthma during the previous year.

The primary outcome variable was asthma symptom control according to the GINA guidelines (well controlled, partly controlled or uncontrolled). Baseline characteristics of patients with well-controlled, partly controlled and uncontrolled asthma were compared using the Kruskal-Wallis test for continuous variables and the chi-squared test for categorical variables. The Jonckheere-Terpstra test for trends was applied if the Kruskal-Wallis test yielded a significant association, and the Mantel-Haenzel test for trends was applied if the chi-squared test yielded a significant association. Univariate logistic regression analyses were performed to assess the association of asthma control with patient characteristics. Before analysis, the 3 asthma control categories were dichotomized in separate analyses as follows: well controlled versus partly controlled and uncontrolled; uncontrolled versus well and partly controlled. In other words, the opposite categories, well-controlled asthma and uncontrolled asthma, were set as separate dependent variables. Independent variables that were significant or almost significant (*p* < 0.10) factors in the univariate models were introduced into the multivariate models. Multivariate logistic regression analyses were then performed using the forward and backward stepping covariate selection procedures. At each step, the criterion for entry was *p* < 0.05 and that for removal was *p* > 0.05. First-order interactions between the covariates were tested. The results are given as odds ratios (ORs) with 95% confidence intervals (95% CIs).

*P*-values less than 0.05 were considered statistically significant. The analyses were performed using IBM SPSS Statistics for Windows (version 25.0, Armonk, NY, USA, IBM Corp.).

## Results

One hundred ninety patients were investigated (Table 1). Their median age (range) was 58 (23–70) years, they had smoked for 20 (10–60) pack-years, and their BMI was 27.5 (16.1–50.3) kg/m^2^. Inhaled corticosteroids (ICS) were used by 179 (94.2%) patients, and leukotriene antagonists were used by 34 (17.9%) patients. One hundred twenty-two (64.2%) of the patients were using both ICS and inhaled long-acting B2-adrenergic (LABA). Eighty-three (44.1%) of the patients were current smokers, and 78 (41.1%) were male.

**Table 1:** Patient characteristics for the whole group of asthmatics who had a smoking history of at least 10 pack-years, and according to asthma symptom control level as per the GINA guidelines

	All subjects (*n* = 190)	Well controlled (*n* = 66)	Partially controlled (*n* = 81)	Uncontrolled (*n* = 43)	*P*-value^*^
Age (years)	58.0 (50.0–65.0)	59.5 (52.0–65.0)	57.0 (49.0–65.0)	58.0 (49.0–64.0)	0.79
Females	112 (58.9)	36 (54.5)	46 (56.8)	30 (69.8)	0.25
BMI (kg/m^2^)	27.5 (24.2–31.1)	26.3 (24.2–30.1)	27.5 (24.3–31.2)	27.9 (23.9–33.3)	0.47
Current smokers	83 (44.1)	23 (35.4)	34 (42.0)	26 (61.9)	0.02 (0.01)^***^
Pack-years	20.0 (15.0–30.0)	20.0 (15.0–30.0)	20.0 (15.0–27.0)	26.0 (15.0–35.0)	0.25
FEV_1_ (% of predicted)	80.5 (71.0–93.0)	84.0 (71.0–94.0)	83.0 (72.0–95.0)	78.0 (67.0–83.0)	0.03 (0.03)^**^
Significant reversibility in FEV_1_	17 (8.9)	5 (7.6)	5 (6.2)	7 (16.3)	0.15
Post-bronchodilator FEV_1_/FVC	0.74 (0.68–0.79)	0.73 (0.68–0.77)	0.75 (0.70–0.81)	0.72 (0.68–0.80)	0.24
Post bronchodilator FEV_1_/FVC < 0.70	52 (27.4)	17 (25.8)	22 (27.2)	13 (30.2)	0.88
Exacerbation during previous year	51 (26.8)	7 (10.6)	27 (33.3)	17 (39.5)	0.001 (< 0.001)^***^
Regular use of inhaled corticosteroid	179 (94.2)	63 (95.5)	77 (95.1)	39 (90.7)	0.53
Regular use of inhaled corticosteroid + long-acting b_2_-agonist (ICS + LABA)	122 (64.2)	42 (63.6)	56 (69.1)	24 (55.8)	0.34

The results are given as the median (interquartile range) or number of patients (percentage)

^*^Kruskal-Wallis test was used for continuous variables, and the chi-squared test was used for comparisons of categorical variables among groups according to asthma control

^**^Jonckheere-Terpstra trend test in parentheses

^***^Mantel-Haenzel trend test in parentheses

Sixty-six (34.7%) patients had their asthma well controlled, 81 (42.6%) had their asthma partly controlled, and 43 (22.6%) were uncontrolled.

The proportions of current smokers (*p* = 0.02) and patients with exacerbations during the previous year (*p* = 0.001) were different among asthma control categories. There was also a difference between the groups in FEV_1_ (% predicted) (Table 1). The proportion of current smokers and patients who had an asthma exacerbation during the previous year decreased when the level of asthma control improved (trend test *p* = 0.01 and *p* < 0.001, respectively). Similarly, FEV_1_ (% predicted) improved as asthma control improved (trend test *p* = 0.03).

The unadjusted analysis of asthma symptom control according to the patient characteristics is shown in Table 2. Current smokers had uncontrolled asthma more often than ex-smokers (31% vs. 15%), OR 2.54 (95% CI 1.25–5.14, *p* = 0.01). Patients who had asthma exacerbations during the previous year had uncontrolled asthma more often than patients without exacerbations (33% vs. 19%), OR 2.17 (95% CI 1.06–4.47, *p* = 0.04). Patients with significant reversibility in spirometry tended to have uncontrolled asthma more often than patients without significant reversibility (41% vs. 21%), OR 2.66 (95% CI 0.95–7.49, *p* = 0.06), and patients with FEV_1_ less than 80% of predicted had uncontrolled asthma more often than patients with FEV_1_ more than 80% of predicted (29% vs. 17%), OR 2.04 (95% CI 1.02–4.08, *p* = 0.04). A smoking history of at least 30 pack-years was related to uncontrolled asthma, OR 1.99 (95% CI 0.98–4.05, p = 0.06). The final multivariate logistic regression model showed that patients with current smoking (OR 2.38, 95% CI 1.14–4.99, *p* = 0.02), smoking history of at least 30 pack-years (OR 2.62, 95% CI 1.21–5.64, *p* = 0.01), exacerbation during the previous year (2.73, 95% CI 1.24–6.04, p = 0.01) or significant reversibility (OR 3.91, 95% CI 1.27–12.09, p = 0.02) had significantly more often uncontrolled asthma. None of the tested interaction terms were significant.

**Table 2:** Asthma symptom control according to patient characteristics. Univariate logistic regression analysis was used to analyse the associations with the asthma control levels ‘Well controlled’ and ‘Uncontrolled’

		Asthma symptom control			Univariate logistic regression analyses					
		Well controlled N (%)	Partly controlled N (%)	Uncontrolled N (%)	Dependent variable is “Well controlled” asthma			Dependent variable is “Uncontrolled” asthma		
					OR	95% CI	P	OR	95% CI	P
Smoking	Ex-smoker (*N* = 105)	42 (40.0)	47 (44.8)	16 (15.2)						
	Current smoker (*N* = 83)	23 (27.7)	34 (41.0)	26 (31.3)	0.58	0.31–1.07	0.08	2.54	1.25–5.14	0.01
Pack-years	< 30 (*N* = 133)	47 (35.3)	61 (45.9)	25 (18.8)						
	≥30 (*N* = 57)	19 (33.3)	20 (35.1)	18 (31.6)	0.91	0.47–1.76	0.79	1.99	0.98–4.05	0.06
Sex	Female (*N* = 112)	36 (32.1)	46 (41.1)	30 (26.8)						
	Male (*N* = 78)	30 (38.5)	35 (44.9)	13 (16.7)	1.32	0.72–2.41	0.37	0.55	0.26–1.13	0.10
Exacerbation^a^	No (*N* = 139)	59 (42.4)	54 (38.8)	26 (18.7)						
	Yes (*N* = 51)	7 (13.7)	27 (52.9)	17 (33.3)	0.22	0.09–0.51	0.001	2.17	1.06–4.47	0.04
ICS	No (*N* = 11)	3 (27.3)	4 (36.4)	4 (36.4)						
	Yes (*N* = 179)	63 (35.2)	77 (43.0)	39 (21.8)	1.45	0.37–5.65	0.59	0.49	0.14–1.75	0.49
ICS + LABA	No (*N* = 68)	24 (35.3)	25 (36.8)	19 (27.9)						
	Yes (*N* = 122)	42 (34.4)	56 (45.9)	24 (19.7)	0.96	0.52–1.79	0.90	0.63	0.32–1.26	0.19
FEV_1_/FVC < 0.70^b^	No(*N* = 138)	49 (35.5)	59 (42.8)	30 (21.7)						
	Yes (*N* = 52)	17 (32.7)	22 (42.3)	13 (25.0)	0.88	0.45–1.73	0.72	1.20	0.57–2.53	0.63
FEV_1_, % of predicted	≥ 80%	40 (39.6)	44 (43.6)	17 (16.8)						
	< 80%	26 (29.2)	37 (41.6)	26 (29.2)	0.63	0.34–1.15	0.13	2.04	1.02–4.08	0.04
Significant reversibility^c^	No (*N* = 173)	61 (35.3)	76 (43.9)	36 (20.8)						
	Yes (*N* = 17)	5 (29.4)	5 (29.4)	7 (41.2)	0.77	0.26–2.27	0.63	2.66	0.95–7.49	0.06

^a^hospitalization and/or oral corticosteroids for asthma during the previous year, ^b^post-bronchodilator, ^c^change in FEV_1_ at least 12% and 200 ml

Having well controlled asthma was not explained by the same patient characteristics as having uncontrolled asthma. Only two significant or almost significant associations were observed in the multivariate logistic regression. Patients with an exacerbation during the previous year had less often well controlled asthma compared to patients without an exacerbation (0.22, 95% CI 0.09–0.51, *p* = 0.001), and currents smokers had less often well controlled asthma compared to ex-smokers (0.58, 95% CI 0.31–1.07, *p* = 0.08) (Table 2).

## Discussion

One third of asthmatics with a smoking history had their asthma well controlled. The proportion is relatively low and suggests that good asthma control among current or ex-smokers is not often achieved in primary care, even in a country like Finland where asthmatics in general do quite well [1].

In a recent survey of 8000 European asthmatics, with only 22.8% of the patients being current smokers, Price et al. [2] found that only 20.1% of asthmatics had their asthma well controlled according to the GINA guidelines. Compared to that, our finding that 34.7% of current or ex-smoking asthmatics had their asthma well controlled was surprisingly high. Most probably this is due to fact that in our study practically all patients were using inhaled corticosteroids regularly, which was not the case in the study by Price et al. In the study by Braido et al. [9], 43.5% of the patients had controlled asthma. In that study 35% of patients were either current or ex-smokers. However, they did not use GINA criteria to define the asthma control level, and therefore their result cannot be directly compared with our result.

Our study included only subjects with a smoking history of at least 10 pack-years, and we cannot therefore make any conclusions regarding comparisons with non-smokers with asthma in Finland. Based on a recent report on a cohort of adult asthmatics in Finland including both smokers and non-smokers, 72% of the subjects had well-controlled asthma defined as an Asthma Control Test (ACT) score of at least 20 points [12]. This is a significantly higher proportion than in our study. Although GINA symptom control and the ACT are not identical, they are quite similar, and this comparison supports previous findings suggesting that asthma symptom control is on average poorer among subjects with a clinically significant smoking history than among others. This is further supported by the same group reporting that in their cohort, asthma symptom control seemed to be worst in those with the heaviest smoking history [13].

From the GOAL study, we learned that it is possible to achieve good asthma control in at least 70% of cases by stepping up asthma medication [14]. More than 94% of our patients were using inhaled corticosteroids (ICS) regularly, and 64.2% of the patients used ICS in combination with long-acting β_2_-agonist (LABA). Nevertheless, only one-third of our patients had their asthma well controlled. This is most likely because our patients had smoked more than 10 pack-years, and 44.1% of our patients were current smokers, and smoking is known to reduce the response to ICS [15, 16]. However, in the current study, using both ICS and LABA increased the likelihood of asthma being well controlled among the subgroup of current smokers with an OR 3.39 (95% CI 1.03–11.20, *p* = 0.04) compared with not using ICS and LABA.

Although 94.2% of our patients used inhaled corticosteroids, 64.2% in combination with LABA, and 17.9% of the patients used leukotriene antagonists, we cannot state that our patients were optimally treated. Namely, as we investigated asthmatics who had smoked at least 10 pack-years, with 27.4% of patients having a post-bronchodilator FEV_1_/FVC < 0.70, suggesting the possibility of asthma-COPD overlap syndrome [11], it is probable that more patients would have had their asthma better controlled if they had also used LAMA. This study is part of a project where we assessed the prevalence of previously undiagnosed asthma-COPD overlap among asthmatic subjects with a smoking history. Therefore, subjects using drugs mostly used to treat COPD at the time of recruitment (LAMA, indacaterol, and roflumilast) were excluded. This was to avoid recruiting subjects with a previous diagnosis of COPD, but since some of the subjects with more severe asthma are treated with LAMA, this criterion may have excluded some subjects with more severe asthma.

We found that uncontrolled patients were current smokers more often than well/partly controlled patients (61.9% vs 39.0%; *p* = 0.009). The finding that current smoking is associated with worse asthma control has also been found by others [6, 8] and may reflect the fact that cigarette smoke is known to reduce the response to ICS in asthmatic patients [15, 16]. Furthermore, there is good evidence that asthma outcome improves in several ways after smoking cessation [3, 5], which is also in line with our observation that ex-smokers had their asthma better controlled than current smokers.

## Conclusions

Asthmatic patients with a smoking history of at least 10 pack-years who are treated in primary health care are often not well controlled. Poorer asthma symptom control was associated with current smoking, history of exacerbations and impaired lung function. Therefore, every attempt should be made to help asthmatics who smoke to quit smoking and to prevent young asthmatics from starting to smoke.

## Supplementary information

**Additional file 1.** Symptom questionnaire, presents an English translation of the symptom questionnaire with criteria used for grading asthma symptom control.

## Data Availability

Due to local legislation on data protection we are not allowed to provide original data on individual level, but on a reasonable request, aggregated data is available from the authors.

## Abbreviations

ACT: Asthma control test
BMI: Body mass index
CI: Confidence interval
COPD: Chronic obstructive pulmonary disease
FEV1: Forced expiratory volume in 1 s
FVC: Forced expiratory volume
GINA: Global initiative for asthma
ICS: Inhaled corticosteroids
LABA: Long-acting beta-2-agonist
LAMA: Long-acting muscarinic antagonist

## References

[1] Haahtela T, Tuomisto LE, Pietinalho A, Klaukka T, Erhola M, Kaila M (2006). A 10 year asthma programme in Finland: major change for the better. Thorax.

[2] Price D, Fletcher M, van der Molen T (2014). Asthma control and management in 8,000 European patients: the REcognise asthma and LInk to symptoms and experience (REALISE) survey. NPJ Prim Care Respir Med.

[3] Perret JL, Bonevski B, McDonald CF, Abramson MJ (2016). Smoking cessation strategies for patients with asthma: improving patient outcomes. J Asthma Allergy.

[4] Polosa R, Thomson NC (2013). Smoking and asthma: dangerous liaisons. Eur Respir J.

[5] Chatkin JM, Dullius CR (2016). The management of asthmatic smokers. Asthma Res Pract.

[6] Pedersen SE, Bateman ED, Bousquet J, Busse WW, Yoxall S, Clark TJ (2007). Determinants of response to fluticasone propionate and salmeterol/fluticasone propionate combination in the gaining optimal asthma controL study. J Allergy Clin Immunol.

[7] Polosa R, Russo C, Caponnetto P, Bertino G, Sarva M, Antic T (2011). Greater severity of new onset asthma in allergic subjects who smoke: a 10-year longitudinal study. Respir Res.

[8] Kämpe M, Lisspers K, Ställberg B, Sundh J, Montgomery S, Janson C. Determinants of uncontrolled asthma in a Swedish asthma population: cross-sectional observational study. Eur Clin Respir J. 2014;1. 10.3402/ecrj.v1.24109 eCollection 2014. 10.3402/ecrj.v1.24109 PMC4629716 26557235

[9] Braido F, Brusselle G, Guastalla D, Ingrassia E, Nicolini G, Price D (2016). Determinants and impact of suboptimal asthma control in Europe: The international cross-sectional and longitudinal assessment on asthma control (liaison) study. Respir Res.

[10] Global Initiative for Asthma (GINA). Global strategy for asthma management and prevention. 2015; Available at: www.ginasthma.org, 2015.

[11] Kiljander T, Helin T, Venho K, Jaakkola A, Lehtimaki L (2015). Prevalence of asthma-COPD overlap syndrome among primary care asthmatics with a smoking history: a cross-sectional study. NPJ Prim Care Respir Med.

[12] Ilmarinen P, Tuomisto LE, Niemela O, Kankaanranta H (2019). Prevalence of Patients Eligible for Anti-IL-5 Treatment in a Cohort of Adult-Onset Asthma. J Allergy Clin Immunol Pract.

[13] Tommola M, Ilmarinen P, Tuomisto LE, Lehtimäki L, Niemela O, Nieminen P, et al. Cumulative effect of smoking on disease burden and multimorbidity in adult-onset asthma. Eur Respir J 2019;54(3):10.1183/13993003.01580-2018. Print 2019 Sep. 10.1183/13993003.01580-2018 31048351

[14] Bateman ED, Boushey HA, Bousquet J, Busse WW, Clark TJ, Pauwels RA (2004). Can guideline-defined asthma control be achieved? The gaining optimal asthma ControL study. Am J Respir Crit Care Med.

[15] Chalmers GW, Macleod KJ, Little SA, Thomson LJ, McSharry CP, Thomson NC (2002). Influence of cigarette smoking on inhaled corticosteroid treatment in mild asthma. Thorax.

[16] Tomlinson JE, McMahon AD, Chaudhuri R, Thompson JM, Wood SF, Thomson NC (2005). Efficacy of low and high dose inhaled corticosteroid in smokers versus non-smokers with mild asthma. Thorax.

