# Supplementary material for: Symptom control among asthmatics with a clinically significant smoking history: a cross-sectional study in Finland
Source: BMC Pulm Med. 2020 Apr 15;20:88. doi: 10.1186/s12890-020-1127-9 (PMC7158041; doi:10.1186/s12890-020-1127-9)
Supplement: Supplementary file 1 — Additional file 1. Symptom questionnaire, presents an English translation of the symptom questionnaire with criteria used for grading asthma symptom control. [file 12890_2020_1127_MOESM1_ESM.docx]

Additional file 1

**Symptom questionnaire**

1. During the last week, has the patient had dyspnoea or cough?
   1. No
   2. Once
   3. Twice
   4. More than twice
2. During the last week, how many times did the patient need reliever medication?
   1. None
   2. Once
   3. Twice
   4. More than twice
3. During the last week, have the patient’s activities, at work or at leisure time, been limited by respiratory symptoms?
   1. No
   2. Yes
4. During the last week, has the patient had any night awakenings due to respiratory symptoms?
   1. No
   2. Yes

Grading asthma symptom control:

- “Well controlled” if all the following criteria are fulfilled:
  - Q1: a-c
  - Q2: a-c
  - Q3: a
  - Q4: a
- “Partly controlled”, if 1 or 2 of the criteria above not fulfilled
- “Uncontrolled”, if 3 or 4 of the criteria above not fulfilled
